# Emerging Sand Fly–Borne Phlebovirus in China 

**DOI:** 10.3201/eid2610.191374

**Published:** 2020-10

**Authors:** Jing Wang, Shihong Fu, Ziqian Xu, Jingxia Cheng, Mang Shi, Na Fan, Jingdong Song, Xiaodong Tian, Jianshu Cheng, Shuqing Ni, Ying He, Wenwen Lei, Fan Li, Heng Peng, Bin Wang, Huanyu Wang, Xiaoqing Lu, Yajun Ma, Guodong Liang

**Affiliations:** Chinese Center for Disease Control and Prevention, Beijing, China (J. Wang, S. Fu, Z. Xu, N. Fan, J. Song, Y. He, W. Lei, Fan Li, H. Wang, G. Liang);; Qingdao University, Qingdao, China (J. Wang, N. Fan, B. Wang, X. Lu);; Shanxi Province Center for Disease Control and Prevention, Taiyuan, China (J. Cheng, X. Tian);; School of Medicine, Sun Yat-sen University, Guangzhou, China (M. Shi);; University of Sydney, Sydney, NSW, Australia (M. Shi);; Wuxiang County Center for Disease Control and Prevention, Wuxiang, China (J. Cheng, S. Ni);; Second Military Medical University, Shanghai, China (H. Peng, Y. Ma)

**Keywords:** amino acid sequences, Bunyavirales, Corfu virus, nucleotide sequences, PCR, polymerase chain reaction, Phlebotomus chinensis, phleboviruses, sand flies, sand fly–borne phlebovirus, Toros virus, vector-borne infections, viruses, Wuxiang virus

## Abstract

We isolated 17 viral strains capable of causing cytopathic effects in mammalian cells and death in neonatal mice from sand flies in China. Phylogenetic analysis showed that these strains belonged to the genus *Phlebovirus*. These findings highlight the need to control this potentially emerging virus to help safeguard public health.

The genus *Phlebovirus* belongs to the order *Bunyavirales*, family *Phenuiviridae* (*1*). Many phleboviruses are sand fly–borne, including sandfly fever Sicilian virus (SFSV), sandfly fever Naples virus (SFNV), and sandfly fever Toscana virus (TOSV), all of which can cause a febrile condition commonly known as three-day fever (*2*). At present, sand fly–borne phleboviruses and their associated diseases are found primarily in countries along the Mediterranean coast, including Italy (*3*), Turkey (*4*), and Cyprus (*5*); there have been no reports of sand fly–borne phleboviruses in China or East Asia (*2*). We describe 17 phlebovirus isolates from sand fly specimens collected in the natural environment of Shanxi Province in central China. 

## The Study 

In June 2018, we collected bloodsucking insects during the evening and night (6:00 PM–7:00 AM) in Wuxiang County (112°26¢–113°22¢E, 36°39¢–37°8¢N), Shanxi Province, China, using Wentaitai MM200 traps (Guangzhou Changsheng Chemical Technology Service Co., https://www.globalsources.com/si/AS/Guangzhou-Changsheng/6008849913119/Homepage.htm#). We classified all specimens according to their morphology under ice bath conditions and stored them in liquid nitrogen until laboratory testing (*6*). We collected a total of 4,069 bloodsucking insects: 3,819 sand flies and 250 mosquitoes. After dividing them into 51 pools (50 mosquitoes or 50–100 sand flies in each pool), we ground insect specimens in minimum essential medium on ice and centrifuged them at 12,000 rpm at 4°C for 30 min. We then processed each homogenate in 2 ways: testing them for the presence of virus with GoTaq Green Master Mix phlebovirus primers (TAKARA, https://www.takarabiomed.com.cn) (*7*) and inoculating them into baby hamster kidney (BHK) 21 cells and *Aedes albopictus* C6/36 cells (*6*). 

**Figure 1:**
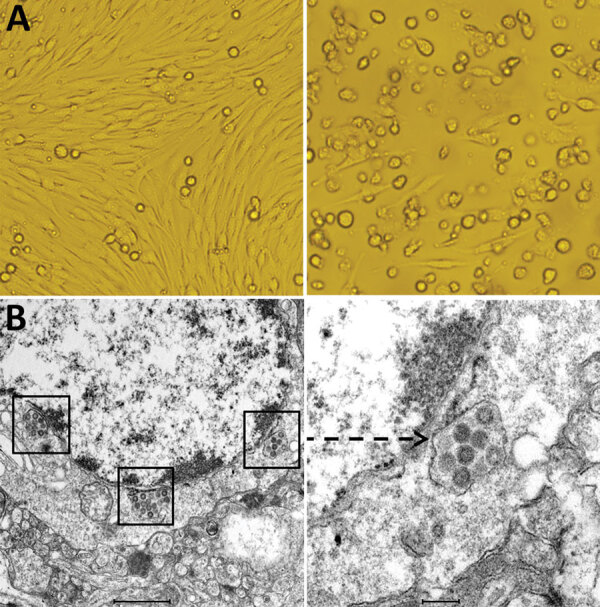
Cytopathogenic effect and electron microscopic morphology of baby hamster kidney 21 (BHK-21) cells infected with phlebovirus, China. A) Left panel shows morphology of BHK-21 cells before inoculation with strain SXWX1813-2; right panel shows morphology 3 days after inoculation (original magnification ×200). BHK-21 cells infected with SXWX1813-2 showed reduced adherence and a large number of rounded and exfoliated cells. B) Left panel shows the viral morphology of SXWX1813–2 on ultrathin slices (scale bar 1μm); right panel shows the enlarged viral particle (indicated by arrow) (scale bar 200nm).

On the third day after inoculation of BHK-21 cells with sand fly specimens, cytopathic effects began to appear (Figure 1). By culturing BHK-21 cells in the presence of supernatant from the sand fly homogenates (Table 1), we obtained 17 viral isolates: 10 strains collected from a sheep pen and 7 from a chicken pen. Pooled supernatants of ground sand flies and viral isolates all showed positive amplification with the phleboviruses primers, and sequencing and analysis revealed that the virus belonged to the genus *Phlebovirus*. We observed no cytopathic effect or *Phlebovirus* genes in C6/36 cells. We used PCR to amplify the cytochrome c oxidase I gene (*8*) and identified *Phlebotomus chinensis* sand flies as the reservoir for each of the 17 phlebovirus-positive pools. 

**Table 1:** Isolation of Wuxiang virus, a new phlebovirus, China

Strain number	No. isolates	Collection place
SXWX1807-1	90	Sheep pen
SXWX1808-2	100	
SXWX1810-1	100	
SXWX1810-2	104	
SXWX1813-1	80	
SXWX1813-2	81	
SXWX1816-1	100	
SXWX1816-4	113	
SXWX1818-1	100	
SXWX1818-2	104	
SXWX1830	13	Chicken pen
SXWX1836-1	100	
SXWX1838-1	90	
SXWX1838-2	81	
SXWX1840-1	100	
SXWX1841-1	100	
SXWX1841-3	100	

To further characterize the phleboviruses isolates, we used a plaque assay 3 times to purify virus from Wuxiang County, Shanxi Province (SXWX-1813-2) and then inoculated it into neonatal mice (*9*), resulting in substantial morbidity and death. The viral titer used in these experiments was 10^8.09^ PFU/mL (4th passage). We observed a large number of virus particles in ultrathin sections of brain tissue of neonatal mice under electron microscopy; the virus particles were uniform spherical particles with diameters of 80–100 nm (Figure 1) (*9*). 

We obtained whole-genome sequences of SXWX1813-2 using a combination of 54 primers covering the viral large (L), medium (M), and small (S) segment genes (Table 2). We used SeqMan (DNAStar, https://www.dnastar.com) for nucleotide sequence splicing, MEGA version 6.0 (https://www.megasoftware.net) for phylogenetic analysis, and Meg alignment (DNAStar) for homology analysis (*6*). 

**Table 2:** Comparison genome sequence length and percent homology of reference viruses to Wuxiang virus, a new phlebovirus isolated in China*

Virus strains	L segment, % homology		M segment, % homology		s segment, % homology			
	RdRp		GP		NSP		NP	
	nt	aa	nt	aa	nt	aa	nt	aa
SXWX1813--2	6,273	2,090	4,089	1,362	783	260	741	246
TORV (213/Turkey 2012)	6,273 (77.1)	2,090 (88)	4,080 (71.9)	1,359 (75.4)	783 (75.2)	260 (84.3)	741 (82.2)	246 (96.4)
TORV (292/Turkey 2012)	6,273 (77.1)	2,090 (88)	4,081 (71.9)	1,359 (75.3)	783 (75.1)	260 (84.3)	741 (81.9)	246 (96)
CFUV (Pa Ar 814/Greece 1981)	6,273 (76.4)	2,090 (88.2)	4,080 (70.7)	1,359 (75.6)	783 (74.1)	260 (83.5)	741 (81.9)	246 (96.4)
SFSV (Ethiopia-2011/Ethiopia 2011)	6,273 (71.6)	2,090 (78.4)	4,026 (62.9)	1,341 (57.2)	NA	NA	741 (73.4)	246 (84.2)
SFSV (U30500)	NA	NA	4,026 (63)	1,341 (56.9)	NA	NA	NA	NA
SFTV (Izmir 19/Turkey 2008)	6,273 (71.4)	2,090 (78.5)	4,026 (62.1)	1,341 (57.1)	804 (63.2)	267 (63.2)	741 (74)	246 (83.8)
DASHV (131/Iran 2011)	6,273 (72.3)	2,090 (79.1)	4,029 (65.2)	1,342 (58.7)	786 (63.9)	261 (60.5)	741 (75.7)	246 (85)
SFSV (J04418)	NA	NA	NA	NA	804 (52.2)	267 (62.5)	741 (74.8)	246 (74.5)
SFSV (I-701735/India 1970)	NA	NA	NA	NA	789 (56.2)	262 (62.8)	741 (75.8)	246 (85.8)
SFSV (R-18/Cyprus 1985)	NA	NA	NA	NA	786 (53.3)	261 (60.5)	741 (73.8)	246 (83.8)
SFSV (RM-09/Cyprus 1985)	NA	NA	NA	NA	789 (53.3)	262 (62.8)	741 (73.5)	246 (83.4)
SFSV (91045I/Iran 1975)	NA	NA	NA	NA	786 (61.8)	261 (60.2)	741 (76.9)	246 (84.6)
SFSV (91025B/Iran 1975)	NA	NA	NA	NA	786 (61.6)	261 (60.5)	741 (76.9)	246 (85)
SFSV (Sabin/Italy/1943)	NA	NA	NA	NA	789 (52.2)	262 (62.5)	741 (74.6)	246 (83.8)
SFSV(Cyprus/Cyprus 2002)	NA	NA	NA	NA	787 (52.7)	261 (62.8)	741 (73.7)	246 (84.6)
SFSV (AJ811547)	NA	NA	NA	NA	804 (51.9)	267 (61.7)	741 (74.5)	246 (83)
RVFV (35/74/South Africa 1974)	6,279 (59.5)	2,092 (55.9)	3,594 (34.1)	1,197 (5.3)	798 (6.3)	265 (24.9)	738 (56.6)	245 (52.8)
SFNV (HM566170)	6,288 (56.7)	2,095 (50.7)	3,972 (31.3)	1,323 (4.8)	930 (5.6)	309 (14.9)	762 (43.9)	253 (41.7)

The complete genome of the SXWX1813-2 virus contains 3 segments (Table 2). The L segment (GenBank accession no. MN454526) is 6,456 nt and contains 1 open reading frame (ORF) encoding an RNA-dependent RNA polymerase (RdRp; 2,090 aa). The M segment (accession no. MN454527) is 4,322 nt and contains 1 ORF encoding a glycoprotein precursor (GP; 1362 aa), which is cleaved into mature N and C glycoproteins. The S segment (accession no. MN454528) is 1,693 nt and contains 2 ORFs encoding nonstructural protein (NSP; 260 aa) and nucleocapsid protein (NP; 246 aa). The S and M segment genes of the other 16 viral strains all had the same nucleotide sequence lengths as those of SXWX1813-2. 

The nucleotide and amino acid sequences of SXWX1813-2 were compared with those of other phleboviruses. L, M, and S segment genes showed the strongest homology with those of Toros virus (TORV) and Corfou virus (CFUV) (*8*). The homology between L segments was 76.4%–77.1% on the nucleotide level and 88.0%–88.2% on the amino acid level and between M segments was 70.7%–71.9% on the nucleotide level and 75.3%–75.6% on the amino acid level. For the NSP gene, the nucleotide homology was 74.1%–75.2% and the amino acid homology was 83.5%–84.3%; for the NP gene, the nucleotide homology was 81.9%–82.2% and the amino acid homology was 96%–96.4% (Table 2). For the M and S gene segments of the other 16 viral strains compared with those of SXWX1813-2, nucleotide sequences were 96.9%–99.8% and amino acid sequences were 97.3%–100% identical. 

**Figure 2:**
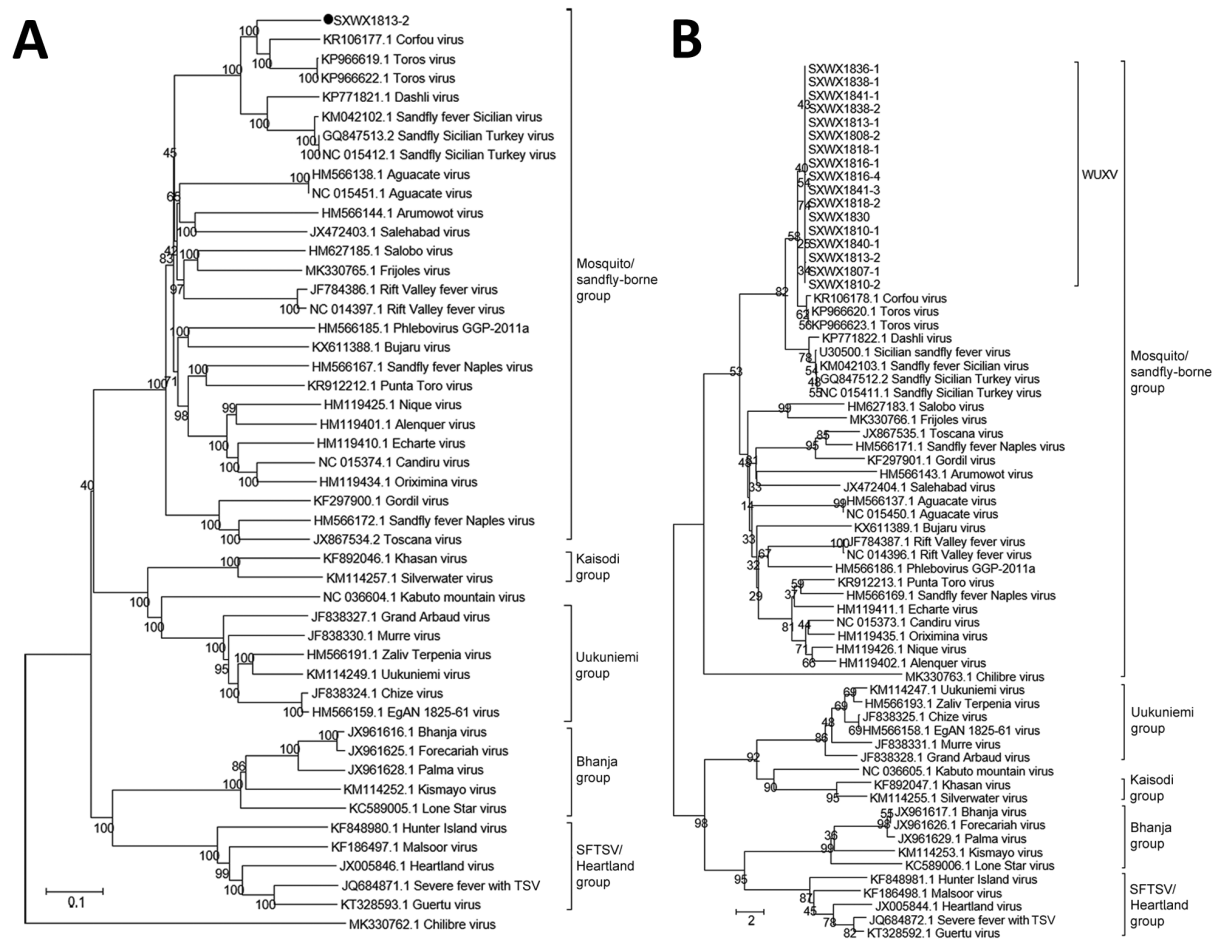
Evolution of nucleotide sequences of the large and medium gene segments of WUXV, a new phlebovirus isolated in China. A) Phylogenetic analysis of nucleotide sequences and molecular genetic evolution analysis of the large gene of WUXV isolate SXWX1813-2 (black dot), and reference isolates. B) Phylogenetic analysis of nucleotide sequences and molecular evolution analyses of the medium genes of 17 WUXV isolates, and reference isolates. MEGA 6.0 (https://www.megasoftware.net) and the neighbor-joining method were used for genetic evolution analysis with 1,000 bootstrap replicates. SFTSV, severe fever with thrombocytopenia syndrome virus; WUXV, Wuxiang virus.

Phylogenetic analysis showed that SXWX1813-2 belongs to the mosquito- and sand fly–borne virus group of the *Phlebovirus* genus. Further analysis showed that SXWX1813-2 is closely related to viruses isolated from sandflies in Turkey (TORV) and Greece (CFUV), forming independent branches. (Figure 2, panel A). The remaining 16 strains isolated from sandflies were all located on the same evolutionary branch as SXWX1813-2 (Figure 2, panel B; Appendix Figures 1, 2). These results suggested that the 17 viral strains isolated from sand flies in this study were a new phlebovirus, which we have named Wuxiang virus (WUXV), the SXWX1813-2 isolate designated as the representative member. 

## Conclusions

Our findings indicate that both sand flies and ticks serve as vectors for phleboviruses, including WUXV, in China. In 2011, severe fever with thrombocytopenia syndrome virus, a tickborne virus known to cause fever and hemorrhaging, was reported in China (*10*). In addition, Guertu virus (*11*) was isolated from *Dermacentor nuttalli* ticks collected in Xinjiang, China. 

To date, both CFUV (*12*) and TORV have been isolated from sand flies collected along the Mediterranean coast in Greece and Turkey. Sand fly–borne phleboviruses, including SFSV, SFNV, and TOSV, are all endemic to the Mediterranean region (*2*). Recently, Drin virus, closely related to CFUV and evolutionarily similar to CFUV and TORV, was isolated in Albania (*13*). Currently, in the taxonomy of the genus *Phlebovirus* CFUV is listed as a tentative species and TORV as an unclassified virus (*8*). Nucleotide- and amino acid–based homology, combined with phylogenetic analysis of phlebovirus genomes, suggests that WUXV is most closely related to TORV and CFUV, with each forming independent branches, indicating that WUXV may be a member of either the Toros-like or Corfu-like viruses. 

*Ph. chinensis* is the dominant sand fly species in China and serves as the primary vector of *Leishmania* in this country (*14*). In our study, we isolated 17 strains of a sand fly–borne phlebovirus, WUXV, from *Ph. chinensis* sand flies, suggesting that the species can also serve as a vector for phleboviruses in China. This finding also suggests the possibility of co-infection with *Leishmania* and phleboviruses. Our finding of phlebovirus in sand flies in China suggests new challenges for controlling a potentially emerging virus to help safeguard public health. 

Appendix Additional information on emerging sand fly–borne phlebovirus in China.
