# Supplementary material for: Emerging Sand Fly–Borne Phlebovirus in China
Source: Emerg Infect Dis. 2020 Oct;26(10):2435–8. doi: 10.3201/eid2610.191374 (PMC7510709; doi:10.3201/eid2610.191374)
Supplement: Appendix — Additional information on emerging sand fly–borne phlebovirus in China. [file 19-1374-Techapp-s1.pdf]

Article DOI: https://doi.org/10.3201/eid2610.191374

Emerging Sand Fly–borne Phlebovirus in China

Appendix

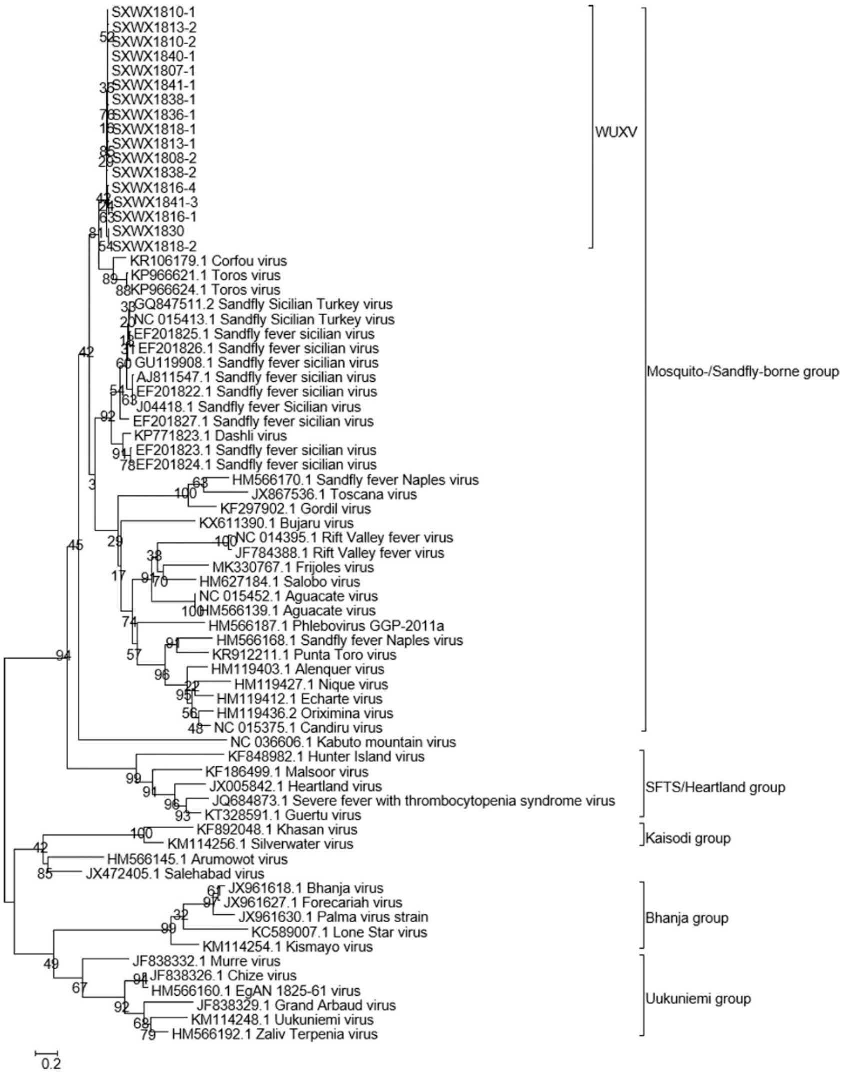

Appendix Figure 1. Evolution of nucleotide sequences of the NS genes of WUXV showing the nucleotide sequencing and molecular evolution analyses of the NS genes of 17 WUXV isolates. MEGA 6.0 and the neighbor-joining method were used for genetic evolution analysis with 1000 bootstrap replicates. SFTSV, severe fever with thrombocytopenia syndrome virus

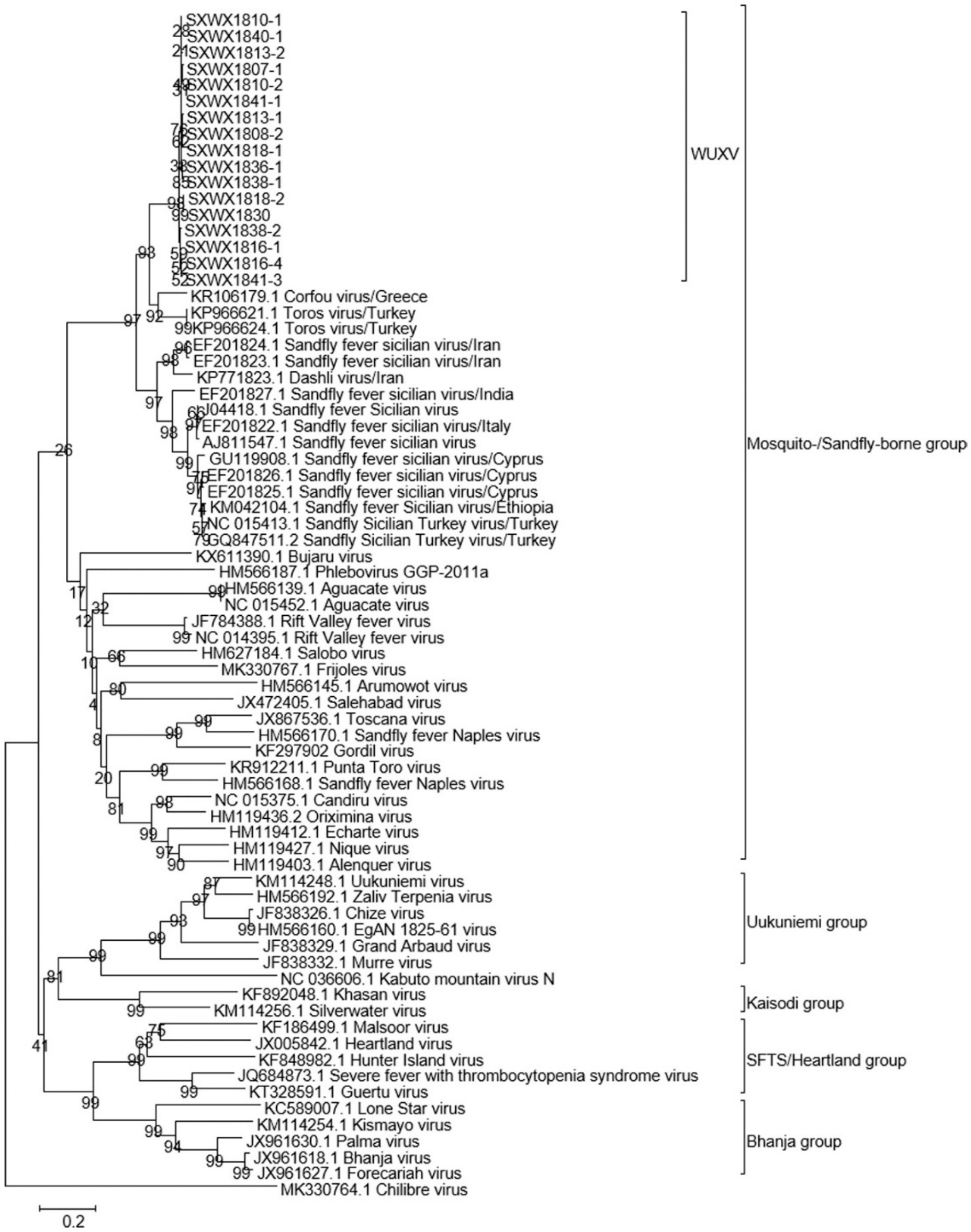

Appendix Figure 2. Evolution of nucleotide sequences of the N genes of WUXV showing the nucleotide sequencing and molecular evolution analyses of the N genes of 17 WUXV isolates. MEGA 6.0 and the neighbor-joining method were used for genetic evolution analysis with 1000 bootstrap replicates. SFTSV, severe fever with thrombocytopenia syndrome virus
